# Design and Preliminary Feasibility Study of a Soft Robotic Glove for Hand Function Assistance in Stroke Survivors

**DOI:** 10.3389/fnins.2017.00547

**Published:** 2017-10-09

**Authors:** Hong Kai Yap, Jeong Hoon Lim, Fatima Nasrallah, Chen-Hua Yeow

**Affiliations:** ^1^Department of Biomedical Engineering, National University of Singapore, Singapore, Singapore; ^2^NUS Graduate School for Integrative Sciences and Engineering, National University of Singapore, Singapore, Singapore; ^3^Department of Medicine, National University of Singapore, Singapore, Singapore; ^4^Queensland Brain Institute, University of Queensland, St. Lucia, QLD, Australia

**Keywords:** soft robotic, hand exoskeleton, rehabilitation, activities of daily living, soft actuators

## Abstract

Various robotic exoskeletons have been proposed for hand function assistance during activities of daily living (ADL) of stroke survivors. However, traditional exoskeletons involve the use of complex rigid systems that impede the natural movement of joints, and thus reduce the wearability and cause discomfort to the user. The objective of this paper is to design and evaluate a soft robotic glove that is able to provide hand function assistance using fabric-reinforced soft pneumatic actuators. These actuators are made of silicone rubber which has an elastic modulus similar to human tissues. Thus, they are intrinsically soft and compliant. Upon air pressurization, they are able to support finger range of motion (ROM) and generate the desired actuation of the finger joints. In this work, the soft actuators were characterized in terms of their blocked tip force, normal and frictional grip force outputs. Combining the soft actuators and flexible textile materials, a soft robotic glove was developed for grasping assistance during ADL for stroke survivors. The glove was evaluated on five healthy participants for its assisted ROM and grip strength. Pilot test was performed in two stroke survivors to evaluate the efficacy of the glove in assisting functional grasping activities. Our results demonstrated that the actuators designed in this study could generate desired force output at a low air pressure. The glove had a high kinematic transparency and did not affect the active ROM of the finger joints when it was being worn by the participants. With the assistance of the glove, the participants were able to perform grasping actions with sufficient assisted ROM and grip strength, without any voluntary effort. Additionally, pilot test on stroke survivors demonstrated that the patient's grasping performance improved with the presence and assistance of the glove. Patient feedback questionnaires also showed high level of patient satisfaction and comfort. In conclusion, this paper has demonstrated the possibility of using soft wearable exoskeletons that are more wearable, lightweight, and suitable to be used on a daily basis for hand function assistance of stroke survivors during activities of daily living.

## Introduction

The ability to perform basic activities of daily living (ADL) impacts a person's quality of life and independence (Katz, 1983; Andersen et al., 2004). However, an individual's independence to perform ADLs is jeopardized due to hand motor impairments, which can be observed in patients with neurological disorders such as stroke. In order to improve hand motor functions in terms of strength and range of motion (ROM) (Kutner et al., 2010), stroke survivors undergo rehabilitation programs comprising repetitive practice of simulated ADL tasks (Michaelsen et al., 2006). Normally, patients undergo rehabilitation exercises in a specialized rehabilitation center under the guidance of physiotherapists or occupational therapists. However, due to increasing patient population, it is foreseen that there will be a shortage of physiotherapists to assist in the rehabilitative process. Thus, there will be comparatively less therapy time, which will eventually lead to a slower recovery process for the patients. Over the past decade, technological developments in robotics have facilitated the rehabilitative process and have shown potential to assist patients in their daily life (Maciejasz et al., 2014). One example of such a device is the hand exoskeleton, which is secured around the hand to guide and assist the movement of the encompassed joints. However, due to the complexity of the hand, designing a hand exoskeleton remains a challenging task.

Traditional hand exoskeletons involve the use of rigid linkage-based mechanisms. In this kind of mechanism, rigid components, such as linear actuators, rotary motors, racks, and pinions as well as rigid linkages are normally involved (Worsnopp et al., 2007; Rotella et al., 2009; Martinez et al., 2010). To assist hand movements that have high degrees of freedom (DOFs), traditional exoskeletons can be incorporated with a substantial number of actuators to achieve the requirement. However, this means that their application is limited due to the increasing bulkiness for higher DOFs. Therefore, these devices are normally restricted in clinical settings and not suitable for performing home therapy. Additionally, their rigidity, weight and constraint on the non-actuated DOFs of the joints pose complications. As a result, the level of comfort and safety of patients is reduced. In view of this, there is an apparent need for the development of exoskeletons that may be used in both clinical and home settings. A lightweight and wearable exoskeleton may allow patients to bring back home to continue daily therapy or to serve as an assistive device for the ADLs.

The development of wearable robotic exoskeletons serves to provide an alternative approach toward addressing this need. Instead of using rigid linkage as an interface between the hand and the actuators, wearable exoskeletons typically utilize flexible materials such as fabric (Sasaki et al., 2004; Yap et al., 2016a) and polymer (Kang et al., 2016), driven by compliant actuators such as cables (Sangwook et al., 2014; Xiloyannis et al., 2016) and soft inflatable actuators (Polygerinos et al., 2015d; Yap et al., 2016c). Therefore, they are more compliant and lightweight compared to the rigid linkage-based mechanism. Cable-driven based exoskeletons involve the use of cables that are connected to actuators in the form of electrical motors situated away from the hand (Nilsson et al., 2012; Ying and Agrawal, 2012; Sangwook et al., 2014; Varalta et al., 2014). By providing actuations on both dorsal and palmar sides of the hand, bi-directional cable-driven movements are possible (Kang et al., 2016). These cables mimic the capability of the tendons of the human hand and they are able to transmit the required pulling force to induce finger flexion and extension. However, the friction of the cable, derailment of the tendon, and inaccurate routing of the cable due to different hand dimensions can affect the efficiency of force transmission in the system.

On the other hand, examples of the soft inflatable actuators are McKibben type muscles (Feifei et al., 2006; Tadano et al., 2010), sheet-like rubber muscles (Sasaki et al., 2004; Kadowaki et al., 2011), and soft elastomeric actuators (Polygerinos et al., 2015b,c; Yap et al., 2015); amongst which, soft elastomeric actuators have drawn increasing research interest due to their high compliance (Martinez et al., 2013). This approach typically embeds pneumatic chamber networks in elastomeric constructs to achieve different desired motions with pressurized air or water (Martinez et al., 2012). Soft elastomeric actuators are highly customizable. They are able to achieve multiple DOFs and complex motions with a single input, such as fluid pressurization. The design of a wearable hand exoskeleton that utilizes soft elastomeric actuators is usually simple and does not require precise routing for actuation, compared to the cable-driven mechanism. Thus, the design reduces the possibility of misalignment and the setup time. These properties allow the development of hand exoskeletons that are more compliant and wearable, with the ability to provide safe human-robot interaction. Additionally, several studies have demonstrated that compactness and ease of use of an assistive device critically affect its user acceptance (Scherer et al., 2005, 2007). Thus, these exoskeletons provide a greater chance of user acceptance.

Table 1 summarizes the-state-of-art of soft robotic assistive glove driven by inflatable actuators. Several pioneer studies on inflatable assistive glove have been conducted by Sasaki et al. (2004); Kadowaki et al. (2011) and Polygerinos et al. (2015a,b,c). Sasaki et al. have developed a pneumatically actuated power assist glove that utilizes sheet-like curved rubber muscle for hand grasping applications. Polygerinos et al. have designed a hydraulically actuated grip glove that utilizes fiber-reinforced elastomeric actuators that can be mechanically programmed to generate complex motion paths similar to the kinematics of the human finger and thumb. Fiber reinforcement has been proved to be an effective method to constrain the undesired radial expansion of the actuators that does not contribute to effective motion during pressurization. However, this method limits the bending capability of the actuators (Figure S1); as a result, higher pressure is needed to achieve desired bending.

**Table 1:** Hand assistive exoskeletons driven by inflatable actuators.

**Device/references**	**Actuators (all pneumatic-driven unless stated otherwise)**	**Weight (g)**	**DOF**	**Operating pressure (kPa)**
Power-Assist Glove/Sasaki et al., 2004	Sheet-like rubber muscles	N.A.	1	500
Power-Assist Glove/Noritsugu et al., 2008	Curved-type rubber artificial muscle	120	1	500
Power-Assist Glove/Kadowaki et al., 2011	Sheet-like rubber muscles	135	4	100
Power-Assist Glove/Toya et al., 2011	Bend-type rubber artificial muscles	180	4	350
Yap et al., 2016c	Elastomeric actuators	200	1	180
ExoGlove/Yap et al., 2015	Elastomeric actuators with variable stiffness	200	5	160
MR-Glove/Yap et al., 2017	Fabric-reinforced actuators	180	5	120
Grip Glove/Maeder-York et al., 2014; Polygerinos et al., 2015a,b,c	Fiber-reinforced actuators (Pneumatic or Hydraulic)	<500	5	345
Nordina et al., 2016	Fiber-reinforced actuators	N.A.	5	300
Haghshenas-Jaryani et al., 2016	Bellow-type soft-rigid hybrid actuators	N.A.	6	165
Exo-Glove PM/Yun et al., 2017	Modular elastomeric actuators	N.A.	5	300
Lobster-inspired Robotic Glove/Chen et al., 2017	Soft actuators with rigid shells	150	5	250
Kline et al., 2005	Rigidizing air bladders	100	1	34
PneuGlove/Connelly et al., 2010; Thielbar et al., 2014	Rigidizing air bladders	N.A.	5	69
Coffey et al., 2014	Rigidizing air bladders	N.A.	1	69
Yap et al., 2016b	Plastic rigidizing actuators	150	5	100

This paper presents the design and preliminary feasibility study of a soft robotic glove that utilizes fabric-reinforced soft pneumatic actuators. The intended use of the device is to support the functional tasks during ADLs, such as grasping, for stroke survivors. The objectives of this study were to characterize the soft actuators in terms of their force output and to evaluate the performance of the glove with healthy participants and stroke survivors. The glove was evaluated on five healthy participants in order to determine the ROM of individual finger joints and grip strength achieved with the assistance of the glove. Pilot testing with two stroke survivors was conducted to evaluate the feasibility of the glove in providing grasping assistance for ADL tasks. We hypothesized that with the assistance of the glove, the grasping performance of stroke patients improved.

Specific contributions of this work are listed as follows:

- Presented fabric-reinforcement as an alternative method to reinforce soft actuators, which enhanced the bending capability and reduced the required operating pressure of the actuators,
- Utilized the inherence compliance of soft actuators and allowed the actuators to achieve multiple motions to support ROM of the human fingers,
- Integrated elastic fabric with soft actuators to enhance the extension force for finger extension,
- Designed and characterized a soft robotic glove using fabric-reinforced soft actuators with the combination of textile materials, and
- Conducted pilot tests with stroke survivors to evaluate the feasibility of the glove in providing functional assistance for ADL tasks.

## Design requirements and rationale

The design requirements of the glove presented in this paper are similar to those presented by Polygerinos et al. (2015a,b,c) in terms of design considerations, force requirements, and control requirements. For design considerations, weight is the most important design criterion when designing a hand exoskeleton. Previous studies have identified the threshold for acceptable weight of device on the hand, which is in the range of 400–500 g (Aubin et al., 2013; Gasser and Goldfarb, 2015). Cable-driven, hydraulic, and pneumatic driven mechanisms are found to be suitable options to meet the criteria. To develop a fully portable system for practical use in home setting, reduction in the weight of the glove as well as the control system is required. The total weight of the control system should not exceed 3 kg (Polygerinos et al., 2015a,b,c). In this work, the criteria for the weight of the glove and control system are defined as: (a) the weight of the glove should be <200 g, and (b) the weight of the control system should be <1.5 kg.

Considering the weight requirement, hydraulic systems are not ideal for this application, as the requirement of a water reservoir for hydraulic control systems and actuation of the actuators with pressurized water will add extra weight to the hand. The second consideration is that the hand exoskeleton should allow fast setup time. Therefore, it is preferable for the hand exoskeleton to fit the hand anatomy rapidly without precise joint alignment. Compared to cable-driven mechanisms, soft pneumatic actuators are found to be more suitable as they allow rapid customization to different finger length. Additionally, they do not require precise joint alignment and cable routing for actuation as the attachment of the soft pneumatic actuators on the glove is usually simple. Therefore, in this work, pneumatic mechanisms were selected. Using pneumatic mechanism, Connelly et al. and Thielbar et al. have developed a pneumatically actuated glove, PneuGlove that is able to provide active extension assistance to each finger while allowing the wearer to flex the finger voluntarily (Connelly et al., 2010; Thielbar et al., 2014). The device consists of five air bladders on the palmar side of the glove. Inflation of the air bladders due to air pressurization created an extension force that extends the fingers. However, due to the placement of the air bladders on the palmar side, grasping activities such as palmar and pincer grasps were more difficult. Additionally, this device is limited to stroke survivors who are able to flex their fingers voluntarily.

In this work, the soft robotic glove is designed to provide functional grasping assistance for stroke survivors with muscle weakness and impairments in grasping by promoting finger flexion. While the stroke survivors still preserve the ability to modulate grip force within their limited force range, the grip release (i.e., hand opening) is normally prolonged (Lindberg et al., 2012). Therefore, the glove should assist with grip release by allowing passive finger extension via reinforced elastic components, similar to Saeboflex (Farrell et al., 2007) and HandSOME (Brokaw et al., 2011). The elastic components of these devices pull the fingers to the open hand state due to increased tension during finger flexion. Additionally, the glove should generate the grasping force required to manipulate and counteract the weight of the objects of daily living, which are typically below 1.07 kg (Smaby et al., 2004). Additionally, the actuators in the glove should be controlled individually in order to achieve different grasping configurations required in simulated ADL tasks, such as palmar grasp, pincer grasp, and tripod pinch. For the speed of actuation, the glove should reach full grasping motion in <4 s during simulated ADL tasks and rehabilitation training.

**Figure 1:**
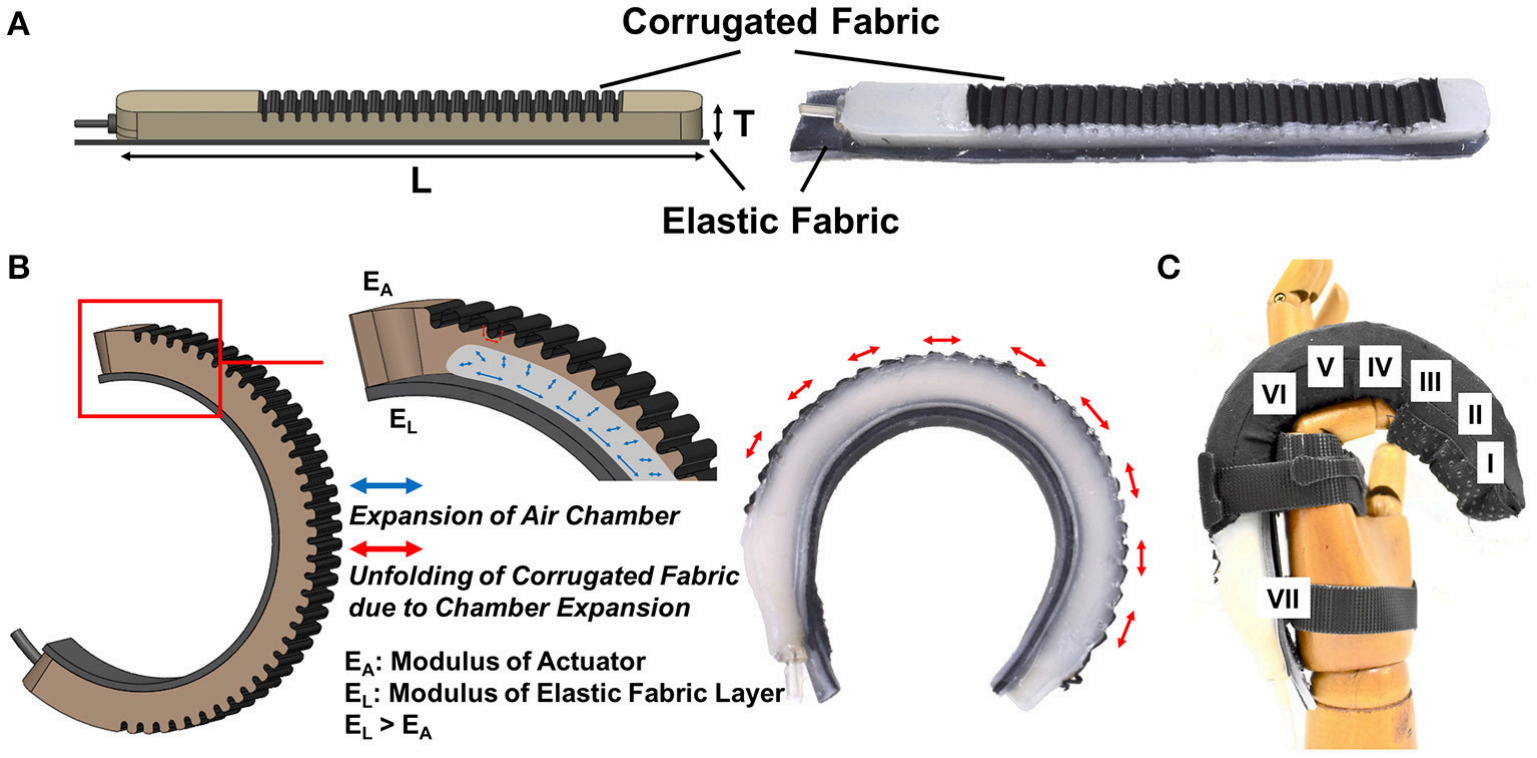
**(A)** A fabric-reinforced soft actuators with a corrugated fabric layer and an elastic fabric later [Actuator thickness, *T* = 12 mm, and length, *L* = 160 mm (Thumb), 170 mm (Little Finger), 180 mm (Index & Ring Fingers), 185 mm (Middle Finger)]. **(B)** Upon air pressurization, the corrugated fabric layer unfolds and expands due to the inflation of the embedded pneumatic chamber. Radial budging is constrained when the corrugated fabric layer unfolds fully. The elastic fabric elongates during air pressurization and stores elastic energy. The actuator achieves bending and extending motions at the same time. **(C)** A bending motion is preferred at the finger joints (II, IV, VI). An extending motion is preferred over the bending motion at the finger segments (I, III, V) and the opisthenar (VII).

For the actuators, we have recently developed a new type of soft fabric-reinforced pneumatic actuator with a corrugated top fabric layer (Yap et al., 2016a) that could minimize the excessive budging and provide better bending capability compared to fiber-reinforced soft actuators developed in previous studies (Polygerinos et al., 2015c,d). This corrugated top fabric layer allows a small initial radial expansion to initiate bending and then constrains further undesired radial expansion (Figure 1). The detailed comparison of the fiber-reinforced actuators and fabric-reinforced actuators can be found in the Supplementary Material.

Upon air pressurization, the top surface of the actuators expands due to the inflation of the embedded pneumatic chamber (Figure 1B). With inextensible layers attached at the bottom of the actuators, bending motion can be obtained due to asymmetrical strain along the length of the actuators. To support a complete hand closure, previous studies, including our group, have developed multi-segment soft actuators that are mechanically programmed to achieve bending and extending motions by controlling the placement of the inextensible bottom layers at different localities along the length of the actuators (Polygerinos et al., 2015b,c; Yap et al., 2017). The bending motion supports finger joint flexion while the extending motion offsets the increased distance due to skin stretching during finger flexion. Bending and extending segments can be pre-designed to conform to the finger anatomy of different patients. This feature demonstrates the high customizability of the soft actuators. However, as the hand dimension varies for different patients, pre-designing actuators with different lengths and controlling the placement of the bending and extending segments for each patient can be time consuming.

Therefore, in this work, we have designed the actuators without pre-programmed bending and extending motions. Instead of controlling the placement of the inextensible bottom layers to achieve bending and extending motions, a single elastic fabric with higher elastic modulus (0.5 N/mm) than the silicone rubber is placed at the bottom of the actuators. With the elastic fabric, the actuators are able to achieve both bending and extending motions (Figure 1B). As the actuators are compliant, they are able to conform to the shape of the object during actuation. A bending motion is preferred at the location with lower impedance, i.e., the finger joints. At the finger segments that possess higher impedance, the bending motion of the actuators is limited and thus the extending motion is preferred (Figure 1C).

## Materials and methods

### Actuator fabrication

A two-part 3D-printed mold is used to fabricate the actuators. A lower-part mold (chamber mold) is used to create a pneumatic chamber inside the actuators, which will inflate upon air pressurization. An upper-part mold (top layer mold) is used to impose a corrugated outer layer for the placement of fabric at the top of the actuators (Figure 1A). The detailed description of the fabrication process as well as the mold dimension can be found in the Supplementary Material.

### Actuator characterization

**Figure 2:**
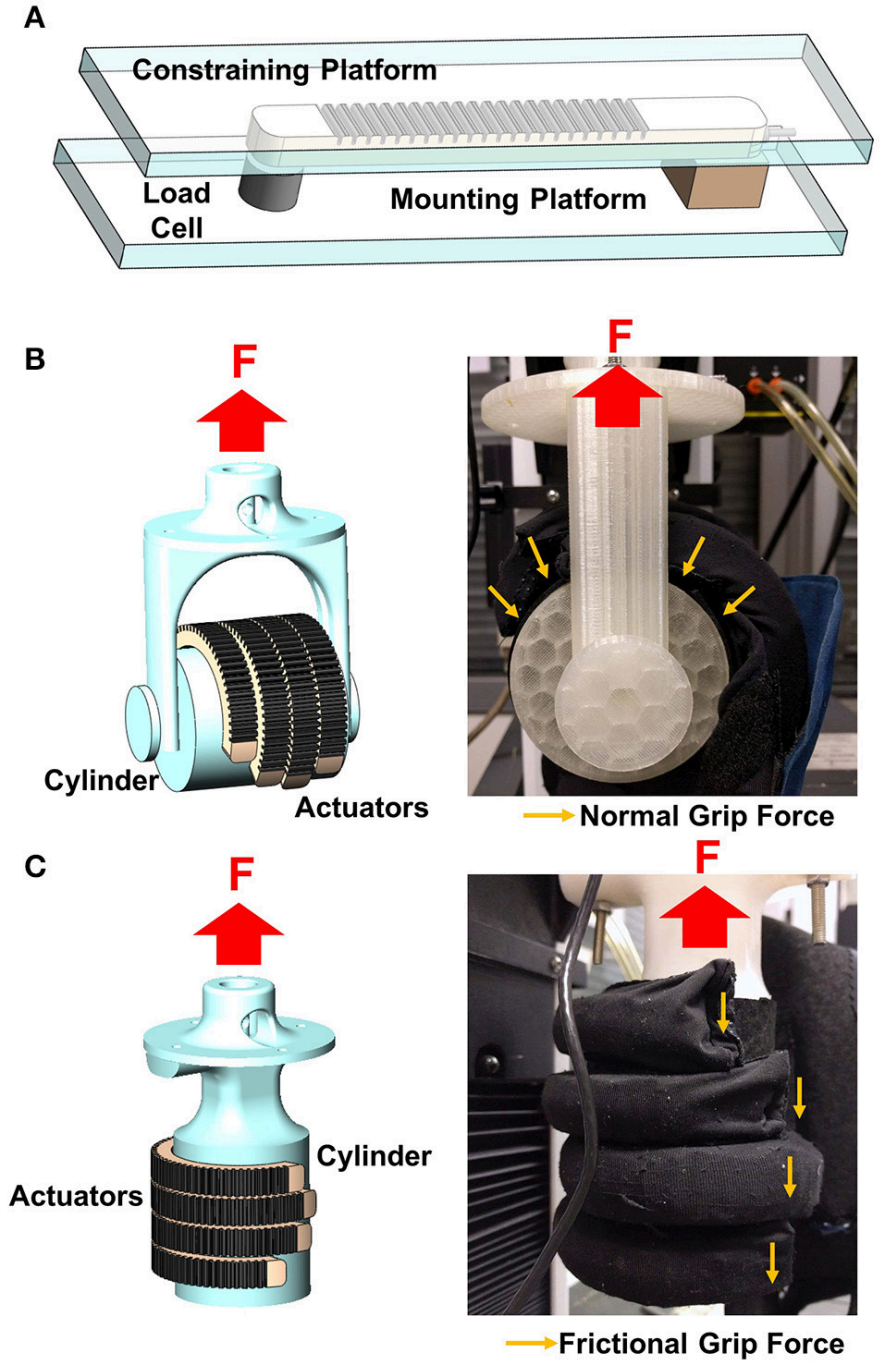
Schematic of the force measurement system for **(A)** blocked tip force, **(B)** normal grip force, and **(C)** frictional grip force measurement.

The actuators were characterized in terms of their blocked tip force and grip force upon pressurization. The blocked tip force exerted by the actuator was measured over increasing pressures using a customized force measurement system (Figure 2A). The system consisted of a compression load cell (FC22, Measurement Specialties Inc., USA) and a mounting platform. The proximal end of the actuator was mounted on the platform and connected to the air source. The distal end of the actuator was in contact with the load cell. A constraining platform was positioned on top of the actuator. During pressurization, the actuator flexed and the tip of the actuator came into contact with the constraining platform, which constrained the height and the curvature of the actuator. This force measurement setup was similar to the setup presented in previous studies (Polygerinos et al., 2015c,d). Constraining the top surface of the actuator minimized the non-linear effects caused by the bending of the actuator when pressurized (Polygerinos et al., 2015d). Thus, this setup could measure the maximum blocked tip force generated by the actuator regardless of the bending angle. The pressure was increased from 0 to 120 kPa. The experiment was repeated three times. Additionally, a theoretical model was adapted from the model presented by Polygerinos et al. (2015d) and Wang et al. (2017) to predict the tip force output of the actuators at this configuration (Figure S4).

For hand exoskeleton application, the force that is of particular interest is the normal grip force (Figure 2B) generated by the gripping of the actuators on an object and the frictional grip force (Figure 2C) that counteracts the weight of that particular object to prevent it from falling. Both the normal and frictional grip forces were measured using a universal testing machine (Model 3345, Instron, MA, USA). Four actuators, which corresponded to four fingers, were pressurized to 120 kPa to enclose and grasp a cylinder, which was of 50 and 75 mm in diameter, in two orientations (i.e., horizontal orientation to measure the normal grip force and vertical orientation to measure the frictional grip force). The cylinder was pulled upward by the Instron at a fixed velocity (8 mm/s) until the cylinder was released from the actuators' grip. The setup was similar to the setup reported in previous literature (Galloway et al., 2016).

Both the normal and frictional grip forces were measured when they were resisting the upward motion of the cylinder. The normal grip force is the sum of the forces applied by four actuators normal to the cylinder surface. The measured force was that resisting the upward motion of the cylinder, which tried to pull the actuators straight from the default bending state (Figure 2B). The frictional grip force is the force exerted by the actuators as the cylinder moves and slides across them (Figure 2C). The experiment was repeated three times and the results were averaged.

### Integration and evaluation of soft wearable robotic glove

**Figure 3:**
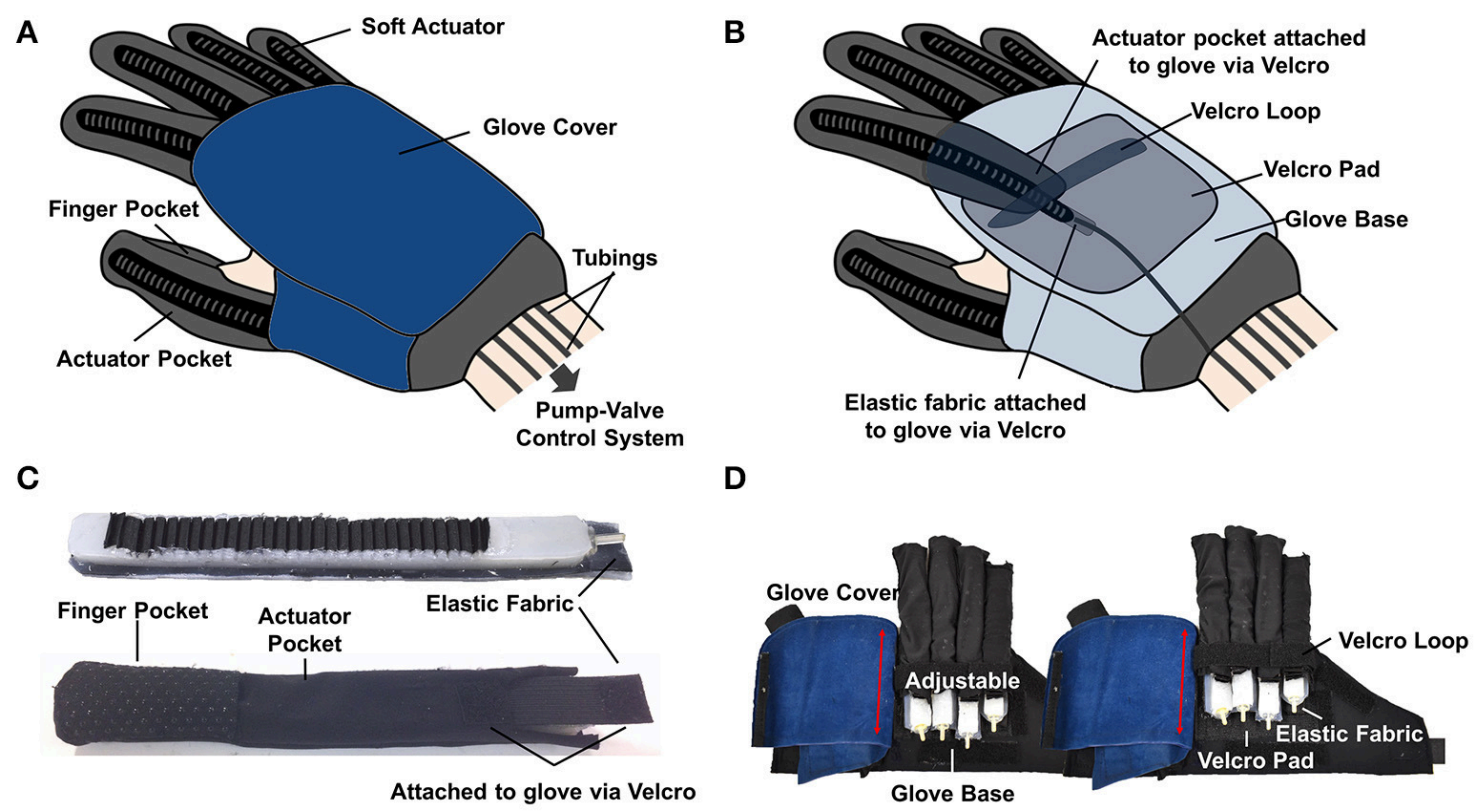
**(A)** Outer view and **(B)** inner view of the soft robotic glove. **(C)** Actuators are inserted into actuator pockets. **(D)** The length of the finger-actuator pockets can be adjusted and attached to the base of the glove via Velcro.

The overall structure of the soft robotic glove is a glove with five finger-actuator pockets attached on the dorsal side (Figure 3A). The actuators are integrated to the glove via the actuator pockets (Figure 3B). Open palm design is adopted for easy donning and doffing of the glove. The glove is secured to the wearer's hand via the finger pockets and a wrist strap. The finger pocket consists of anti-slip material to enhance the grip strength. To wear the glove, the wearer just needs to insert the fingers into the finger pockets and secure the glove to the wrist via the wrist strap. The glove base serves as a compliant interface between the actuators and the human hand, providing minimal mechanical impedance to the finger motion when it is being worn and ensuring kinematic transparency.

The actuators can be easily inserted into the actuator pockets, which are made from stretchable lycra fabrics (Figure 3C). The lycra fabrics serve as second constraining layers for the actuators, which further prevent over expansion of the actuators. Each actuator is isolated with respect to the others. The assistance of each finger can be achieved independently, which allows execution of different simulated ADL tasks. The length of the finger-actuator pockets can be adjusted and attached to the base of the glove via Velcro, in order to accommodate different finger lengths (Figure 3D). Both the Velcro loop and the glove cover constrain the bending movement of the actuators at the proximal part of the hand (Figure 3D).

The total weight of the glove is ~180 g, which is much lower than the design requirement. The thickness of the glove (including the actuators) and the width of each finger-actuator pocket is <2 cm. Additionally, as the actuators work under air pressure, inflation of the actuators does not add a significant amount of extra weight to the hand, as compared to hydraulically actuated actuators.

### Pump-valve control system

**Figure 4:**
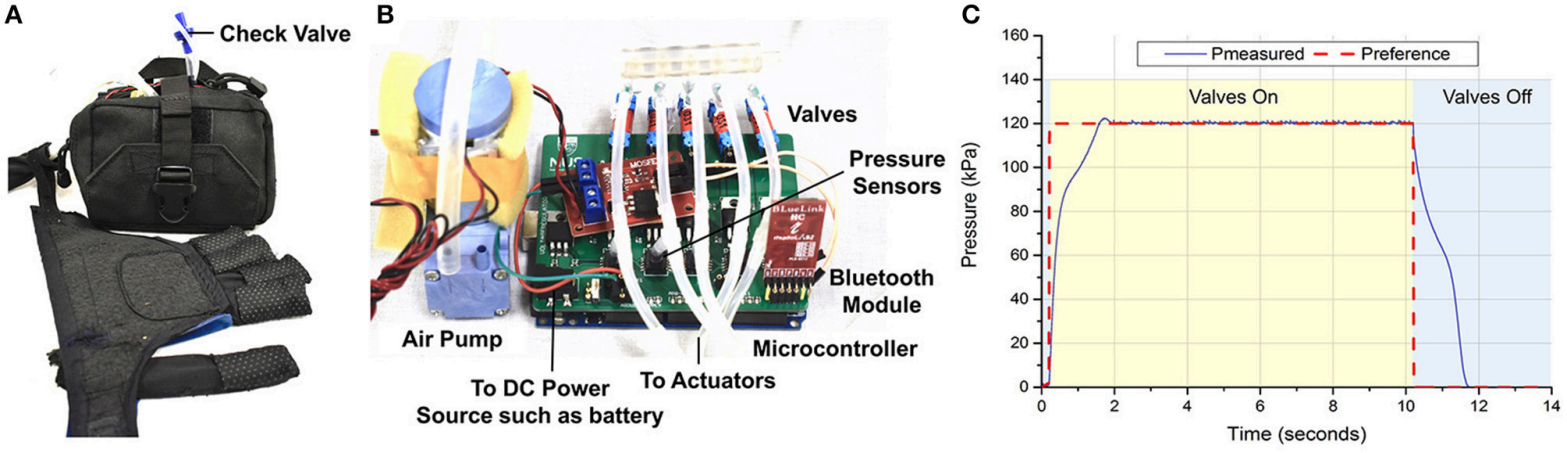
**(A)** Pump-valve control system integrated into a waist belt pack. **(B)** Inner view of the control system. **(C)** Step response of the controller implemented on the pump-valve control system.

To realize isolated control of each actuator, a pump-valve control system was assembled and integrated into a portable waist belt pack (Figure 4A). The control system consisted of a a microcontroller (Arduino Mega, Arduino), a miniature diaphragm pneumatic pump (D737-23-01, Parker, USA), five miniature solenoid valves (X-Valve, Parker, USA), and five air pressure sensors (MPX5500DP, Freescale, USA) (Figure 4B). The microcontroller regulated the measured air pressure (P) to track the desired pressure (P_ref_) and used pulse width modulation (PWM) to control the activation and deactivation of the valves and pump based on the readings of the pressure sensors. The control system could be powered by a 12 V rechargeable lithium polymer battery (DC12300, China). The total weight of the control system was ~1.26 kg, which was lighter than the control system (3.3 kg) presented by Polygerinos et al. (2015c).

A Proportional Integral Derivative (PID) control algorithm was used to ensure that the measured air pressure (P) of the actuators was close to desired air pressure (P_ref_). The PID control scheme was implemented on the microcontroller with a sampling frequency of 100 Hz. The valves have a nominal response time of 20 ms and a PWM frequency of 50 Hz was used. The control parameters were selected as K_p_ = 10, K_i_ = 0.7, and K_d_ = 0.1. The desired pressure P_ref_ was set at 120 kPa, which was the pressure that corresponded to full finger flexion based on the results from ROM test. The actuator for the index finger was pressurized from 0 to 120 kPa. The control loop was tested in a step response experiment (Figure 4C). The closed-loop system had a rise time of 1.36 s and a settling time (5%) of 1.63 s. The closed-loop system bandwidth was found to be 0.342 Hz (Figure S5).

### Glove-assisted range of motion

Five healthy participants (3 males, 2 females, age: 26.2 ± 2.4 yr) were recruited in order to evaluate the performance of the glove in terms of its assisted-finger ROM and grip strength. All participants gave written informed consent in accordance with the Declaration of Helsinki. The study protocol was approved by the Institutional Review Board of the National University of Singapore.

Two sessions were conducted for each participant. In the first session, which consisted of three active trials, the participants were instructed to close and open their right hand with the presence of the glove. The length of the finger-actuator pockets was adjusted to fit the finger length of the specific subject. In the second session, which consisted of three glove-assisted trials, the participants were instructed to relax their muscles and not to exert any voluntary finger action. The hand closing and opening were assisted by the actuation of the soft actuators. EMG signals of the finger flexors and extensors were recorded during the trials to identify whether the subject was exerting voluntary control of the hand during glove-assisted trials.

The assisted-finger ROM and the EMG data were obtained through the test method reported in our previous study (Yap et al., 2016c). Additional details regarding the test method are reported in the “Test Method for Glove-assisted ROM” section of the Supplementary Material.

### Glove-assisted grip strength

**Figure 5:**
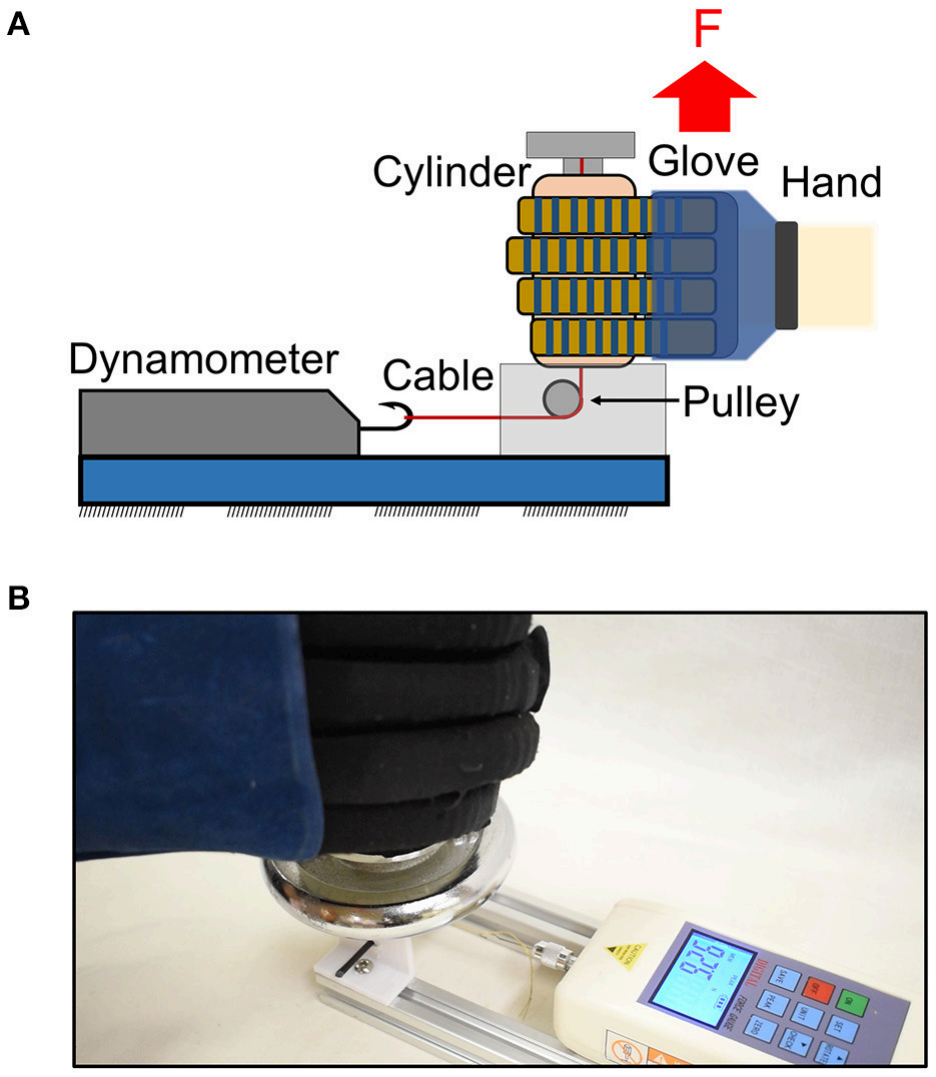
**(A)** Schematic and **(B)** the real setup of a portable setup with dynamometer to measure the glove-assisted grip strength. Maximum frictional grip force was recorded before the cylinder released from the actuators' grip.

A portable setup with a digital dynamometer (HF-50, China) was developed to measure the glove-assisted grip strength of the participants (Figure 5A). Upon pressurization to 120 kPa, the glove assisted participants with grasping a cylinder (50 and 75 mm in diameter) (Figure 5B). The participants were instructed to not exert voluntary force and hence, the grasping of the cylinder would be solely due to the force generated by the inflated actuators. The cylinder was tied to a Kevlar cable attached to the dynamometer. As the participants grasp and lift the cylinder upwards, the cylinder would be slowly released from the actuators' grip, when the tension in the cable became larger than the frictional force applied by the actuators (Figure 5C). This setup allowed the measurement of frictional grip force applied by the actuators, with the glove worn by the participants. The setup and the mechanism of the slip of the cylinder from the hand are similar to those reported in previous literature (Westling and Johansson, 1984; Johansson and Westling, 1988; In et al., 2017).

### Pilot test with stroke survivors

A pilot test that evaluated the feasibility of the glove in providing hand grasping assistance for ADL tasks was performed on two stroke survivors. All participants gave written informed consent in accordance with the Declaration of Helsinki. The study protocol was approved by the NHG Domain Specific Review Board. The subject demographics are shown in Table 2.

**Table 2:** Stroke patients' demographic data.

**Sex (age)**	**Time post stroke (months)**	**Type of stroke**	**Affected side**	**Fugl-meyer motor assessment (Upper extremity)**	**Modified ashworth scale**
F (50)	13	Infarct	Left	4	1
M (40)	10	Infarct	Left	5	1+

The patients were instructed to use their affected hand to grasp an empty water bottle (Weight: 20 g, Diameter: 60 mm) and a tin can (Weight: 454 g, Diameter: 75 mm) typically used in Jebsen Hand Function Tests (Beebe and Lang, 2009). The same task was repeated having the patients wear the glove. The task was considered successful if the patient was able to grasp, lift, and put down the object. The participants were allowed to use their non-paretic hand to support their paretic forearm if they could not lift their paretic arm. The time taken to complete the tasks (grasp, lift, and put down the bottle or can) was recorded. The maximum time allowed to complete the task was 90 s. The experiment was repeated three times for each object and the results were averaged across six trials for each patient in each condition (i.e., with and without glove assistance).

In this work, we employed a straightforward button control strategy to control the glove. A phone application with three virtual buttons, (namely grasping, pinching, and tripod pinching) was built to interface with the control system through wireless Bluetooth communication (Figure S7). The patients were instructed to click the virtual buttons using their non-paretic hand to activate the glove for grasping activities. To deactivate the glove, the patients just simply clicked the specific activated button again.

After the test, the patients' feedback on the device was obtained. The patients were instructed to fill in the Usefulness-Satisfaction-and-Ease-of-use questionnaire (USE) (Lund, 2001) and a questionnaire focusing on comfort level, desire to use, and desire to purchase the device. Both questionnaires use a seven-point Likert rating scale that focuses on the experience and feedback of the system.

## Results

### Blocked tip force output

**Figure 6:**
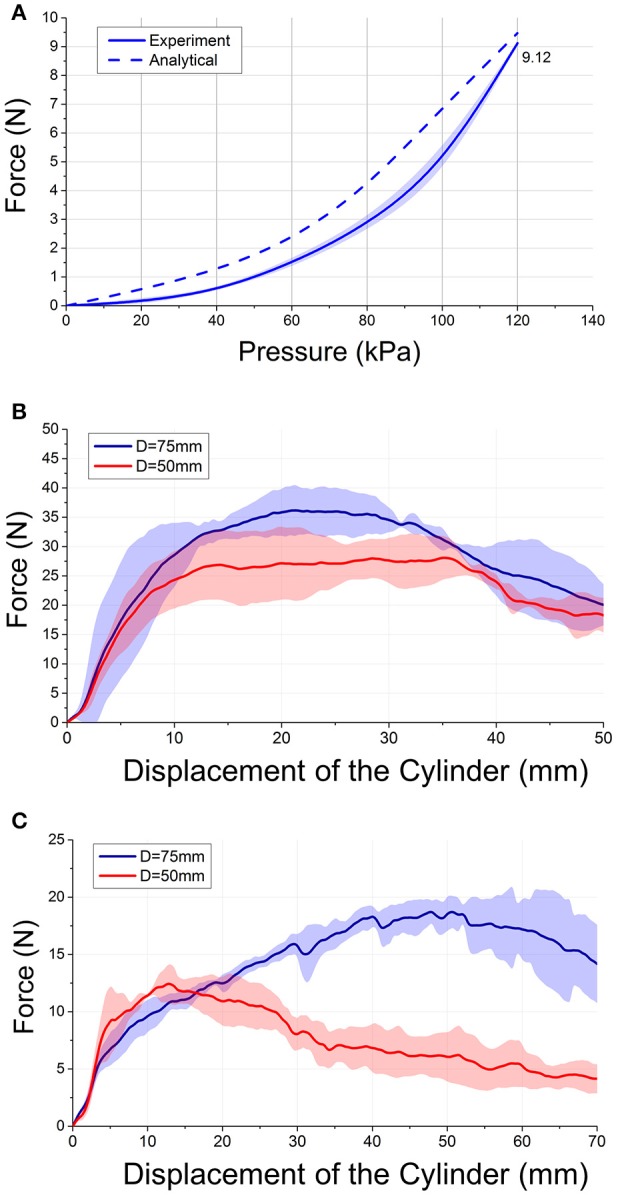
**(A)** Experimental and theoretical blocked tip force values over increasing pressures. **(B)** Normal and **(C)** frictional grip force output-displacement of the cylinder relationships of four actuators gripping cylinder with diameter *D* = 75 mm and *D* = 50 mm at a fixed pressure (120 kPa).

The average values of the experimental force and the force obtained from a theoretical model were compared and shown in Figure 6. The force increased with increased pressure (Figure 6A). The soft actuators could generate a maximum tip force of 9.12 N at 120 kPa. Similarly, the force values obtained from the theoretical model increased with increased pressure, with a tip force value of 9.47 N at 120 kPa.

### Normal and frictional grip force output

Figures 6B,C plotted the force (normal/frictional grip force)—displacement of the cylinder (distance traveled by the cylinder from initial position) characteristics of the force measurement scenario. The average values of the normal and frictional grip force applied by the actuators are shown in Figures 6B,C. The results showed that four actuators could generate a normal grip force of up to 36.2 ± 4.5 N (Diameter: 75 mm) and 28.1 ± 3.6 N (Diameter: 50 mm) (Figure 6B). The actuators could generate a frictional grip force of up to 18.7 ± 0.9 N (Diameter: 75 mm) and 12.4 ± 1.7 N (Diameter: 50 mm) (Figure 6C). The normal and frictional grip forces decreased with decreased cylinder diameter. As the objects of daily living do not weigh more than 1.5 kg (Matheus and Dollar, 2010), the frictional force was found to be sufficient to lift most of the objects.

### Glove-assisted range of motion

**Figure 7:**
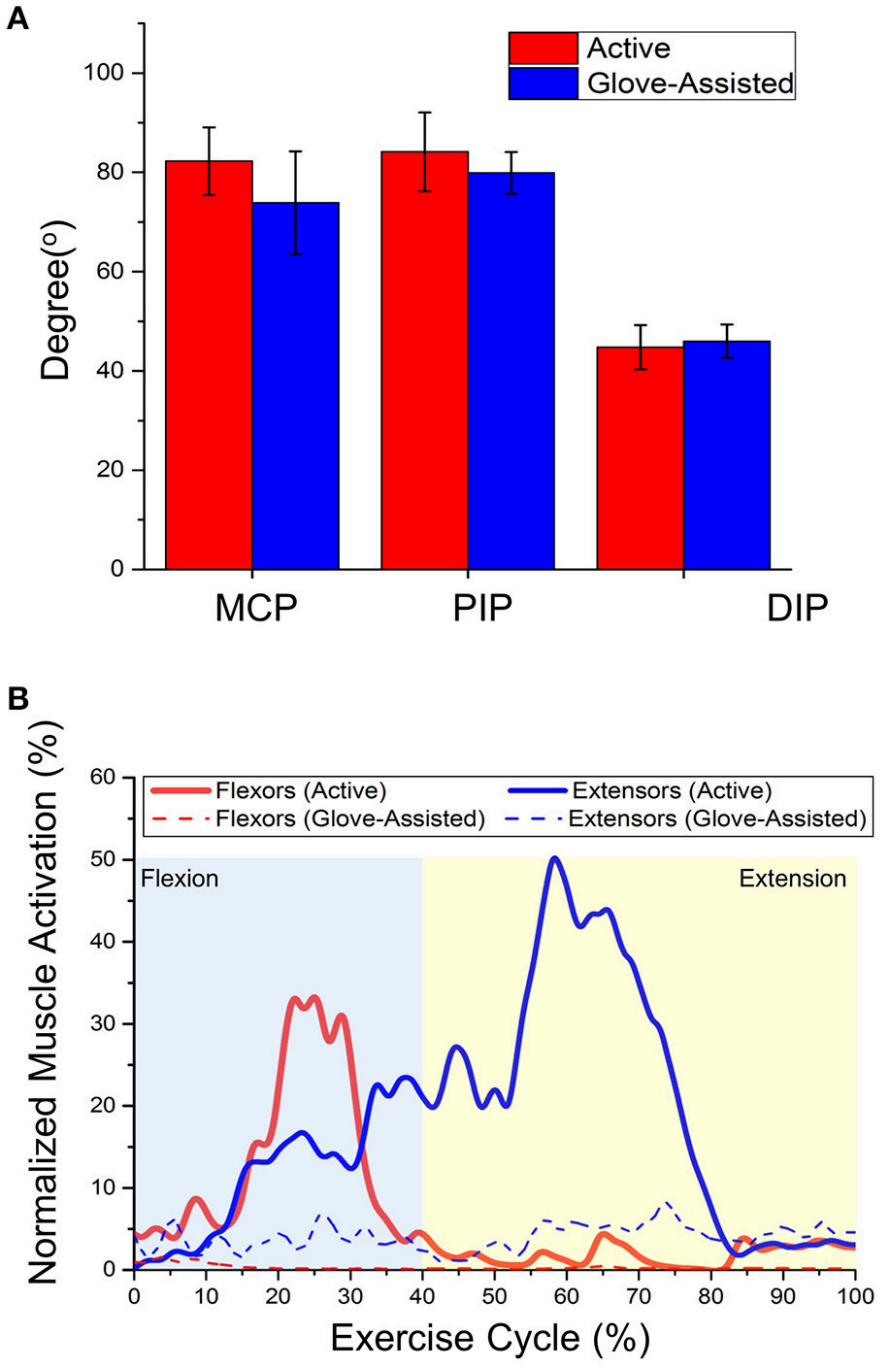
**(A)** Averaged range of motion of individual finger joints of five participants during active and glove-assisted trials. MCP, Metacarpophalangeal joint; PIP, Proximal interphalangeal joint; DIP, Distal interphalangeal joint. **(B)** Averaged muscle activation profiles of finger flexors and extensors of one representative subject (Subject 2) during active and glove-assisted trials. Exercise Cycle, Hand closing followed by opening.

During active trials, the maximum achievable joint angles were 82.3 ± 6.8°, 84.1 ± 7.9°, and 44.8 ± 4.4° at MCP, PIP, and DIP joints. The sums of finger joint angles during active trials were measured to be ~211°. During glove-assisted trials, the maximum achievable joint angles were 73.9 ± 10.4°, 79.9 ± 4.2°, and 46.0 ± 3.3° at MCP, PIP, and DIP joints. The sums of finger joint angles were measured to be ~200° (Figure 7A). Statistical analysis was conducted using the non-parametric Wilcoxon's Signed-Rank tests. The tests showed no significant difference between active and glove-assisted trials (*p* = 0.38 for MCP joint, *P* = 0.32 for PIP joint, and *P* = 1.25 for DIP joint).

### Muscle activation

The muscle activation signals showed clear differences between active and glove-assisted trials (Figure 7B). During the glove-assisted trials, the amplitude of the normalized EMG signal confirmed that the participants exerted minimal muscle effort. Therefore, we concluded that the participants exerted minimal voluntary movement during the glove-assisted trials. The hand closing and opening motions were completely assisted by the glove.

### Glove-assisted grip strength

When the glove was worn by the participants, the glove could generate a frictional grip force up to 8.4 ± 1.8N (Cylinder diameter: 75 mm) and 5.8 ± 1.7 N (Cylinder diameter: 50 mm) to counteract the weight of an object. Statistical analysis was conducted using the Wilcoxon's Signed-Rank tests. The frictional grip force decreased with decreased cylinder diameter. Significant was concluded (*P* = 0.03).

### Simulated ADL tasks

**Figure 8:**
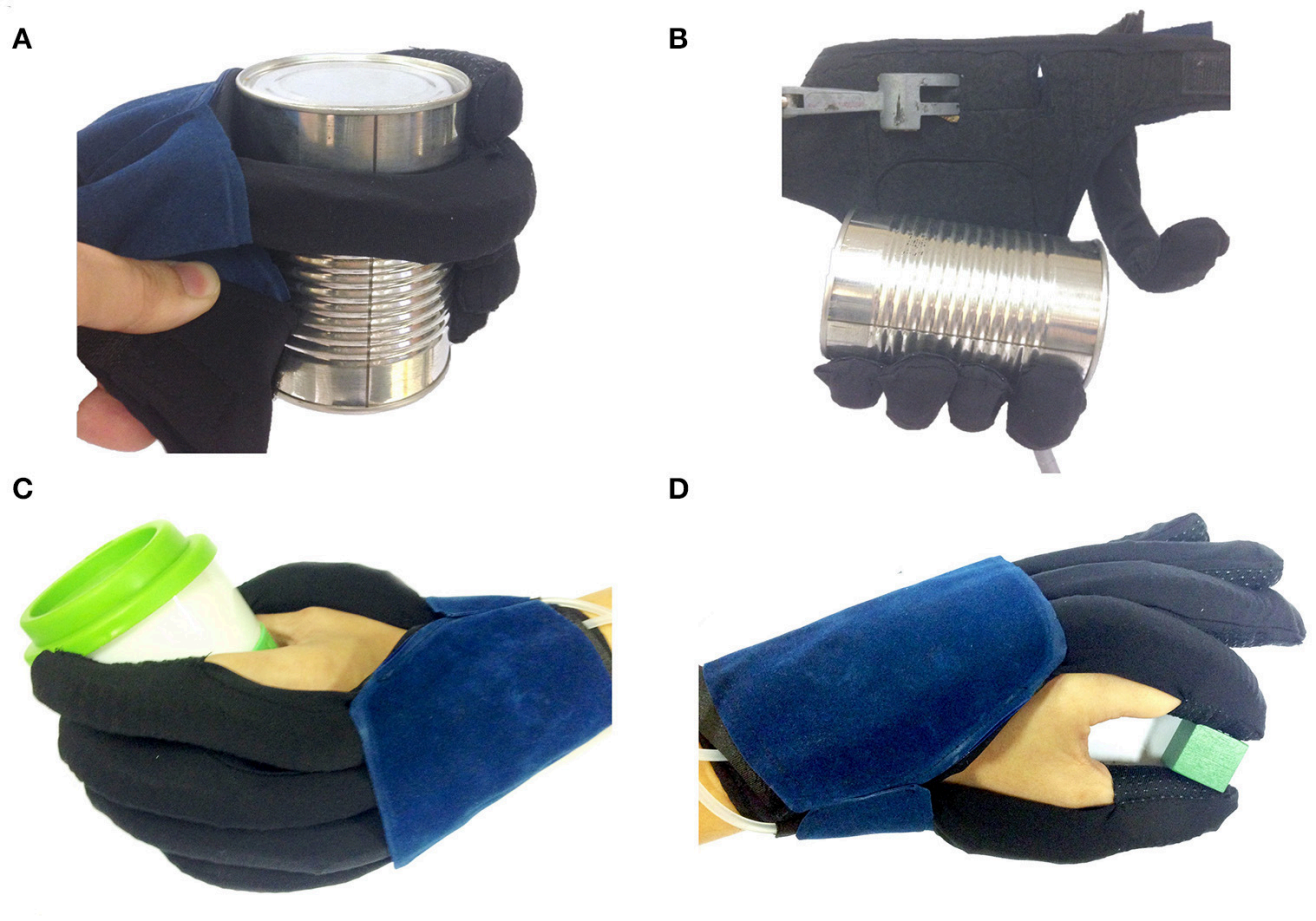
The glove generated sufficient force to lift a 454 g-weighted can in the **(A)** vertical and **(B)** horizontal orientation. **(C)** Palmar grasp, **(D)** Pincer grasp achieved with the assistance of the glove.

Figure 8 demonstrates the ability of the glove to hold and lift a 454 g-weighted tin can in vertical (Figure 8A) and horizontal (Figure 8B) orientation. Additionally, we demonstrated that the glove was also able to assist with palmar grasp and pincer grasp that are required for simulated ADL tasks, such as grasping and pinching (Figures 8C,D).

### Pilot test with stroke survivors

**Figure 9:**
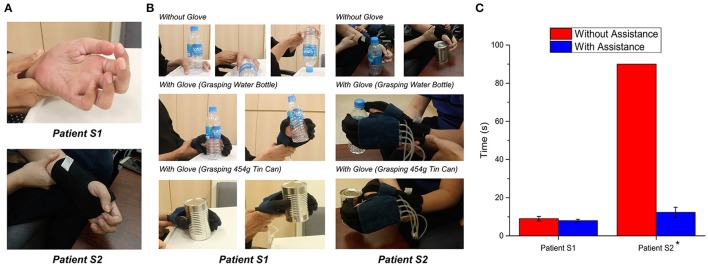
Evaluation of the glove with stroke patient S1 and S2. **(A)** Hand conditions of patients. **(B)** Comparison of grasping performances with and without assistance of glove. **(C)** Time taken to complete the grasping tasks (*n* = 6 for each patient; *P* = 0.06 for S1, *P* = 0.02 for S2, paired *t* test, ^*^p < 0.05.). The actuators of the glove were pressurized at 120 kPa.

Both patients were able to activate the glove using the phone application with virtual buttons (Figure 9). Without the assistance of the glove, patient S1 was able to finish the tasks in an average time of 9.0 ± 1.4 s (Figure 9B). When patient S1 was grasping and lifting the water bottle, the patient could not grasp the bottle effectively and the bottle toppled. With the assistance of the glove, patient S1 could finish the tasks in an average time of 8.0 ± 0.7 s (Figure 9C).

The hand of the patient S2 was slightly clenched. As a result, the patient could not finish the tasks within 90 s as the patient could not grasp both objects (Figure 9B). The patient could finish the tasks in an average time of 12.3 ± 2.7 s with the assistance of the glove (Figure 9C).

Statistical analysis was conducted using the non-parametric tests. Due to poststroke heterogeneity and small sample size, Wilcoxon's Signed-Rank tests on individual participant data were used to determine each patient's improved time-taken to complete the task with the assistance of the glove. Significance was concluded on patient S2 (*p* = 0.02). Although significance was not concluded on patient S1 (*p* = 0.06), we could still observe that patient S1 could grasp the bottle more effectively with the assistance of the glove. Therefore, as hypothesized, the grasping performance of stroke patients improved with the assistance of the glove.

For patients' feedback, the mean and standard deviation (SD) of the questionnaire parameters are presented in Table 3. Generally, the patients reported scale of at least five out of seven-point Likert rating.

**Table 3:** Questionnaire parameters and relative ratings of the USE questionnaire and a questionnaire focusing on comfort level, desire to use, and desire to purchase the device.

**Parameters**	**Mean (SD)**
**USE QUESTIONNAIRES**	
Usefulness	5.9 ± 0.3
Ease of use	6.4 ± 0.4
Ease of learning	6.6 ± 0.2
Satisfaction	6.6 ± 0.5
**PATIENTS' FEEDBACK**	
Comfort level	6.0 ± 1.4
Desire to use	6.5 ± 0.7
Desire to purchase	5.0 ± 1.4

*SD, standard deviation*.

## Discussion

Compared to the fiber reinforced actuators developed for soft robotic glove application in previous studies, which operate at the pressure range of 275–375 kPa (Polygerinos et al., 2015c), the fabric-reinforced soft actuators designed in this study required a lower range of operating pressures to achieve similar force output. With this advantage, we can select pumps and valves that operate at a lower pressure range and consume less power, which will lead to a more portable system.

Compared to the frictional grip force applied by the actuators when the glove was not worn (section Normal and Frictional Grip Force Output), the frictional grip force reduced when the glove was worn by the participants (section Muscle Activation). This might be due to the joint stiffness of the participants that imposed an opposing torque that the actuators needed to overcome during finger flexion. Nevertheless, the force generated was considered sufficient to grasp and achieve most of the ADLs (Matheus and Dollar, 2010; Polygerinos et al., 2015c). The frictional grip force is dependent on the object size, which can be observed from the grip force experiment. The experiment showed that the force output decreased with smaller cylinder diameter. This was because the force exerted by the actuators changed with the distance (i.e., the force decreased with increasing actuator bending angle). Additionally, the frictional grip force is also affected by the friction coefficient of the surface of the object and the anti-slip material of the finger pockets on the glove. The friction coefficient between the anti-slip material on the finger pocket and the surface of the cylinder could be estimated from the relationship between normal grip force and frictional grip force. In the measurement scenario described in this work, the coefficient was estimated at between 0.4 and 0.5.

The average functional ROM of the hand, which are sufficient to perform more than 90% of daily functional activities, at MCP, PIP, and DIP are 61, 60, and 39°, respectively (Hume et al., 1990). Additionally, as the actuators designed in this study are able to extend to offset the increased distance due to skin stretching during finger flexion, the reported assisted ROM at MCP is higher than the assisted ROM reported in our previous study (Yap et al., 2016c). The EMG experiment confirmed that the participants exerted no voluntary effort during the glove-assisted trials and the hand grasping action was completely assisted by the glove. Therefore, our results demonstrated that the glove could achieve sufficient ROM and provide assistance with grasping motions typically found in ADL, in the absence of voluntary muscle control.

The choice of pneumatic over hydraulic allows the control system to be more portable and lightweight than the control system presented by Polygerinos et al. (2015b,c), as it does not require a water reservoir. However, the limitation of the pneumatic system is that it suffers from slow dynamics due to slow valve discharging speed and slow response times when depressurizing, as presented in previous related works on soft robotic gloves (Polygerinos et al., 2015c; Yap et al., 2017). In this work, the closed-loop system bandwidth of the control system was found to be 0.342 Hz. The value (0.342 Hz) is similar to the bandwidth of the control system presented by Polygerinos et al. (0.25 Hz). Although the bandwidth was less than the common bandwidth of rigid linkage-based and cable-driven hand exoskeletons (Agarwal et al., 2015; Xiloyannis et al., 2016), it can be considered sufficient for patients to conduct functional tasks for ADLs. The valves used in current system could be replaced with valves with faster flow rate in the future to increase the bandwidth of the actuators.

The limitation of the previous soft actuator design is that the actuators apply forces to flex the finger upon air pressurization. Upon depressurization, the actuators assist finger extension passively with the elastic properties of the actuators and the elastic textile materials of the actuator pockets (Polygerinos et al., 2015b,c; Yap et al., 2016a, 2017). The extension force might not be enough for patients with hypertonicity and increased finger flexor tone. In this work, the bottom elastic fabric layer is able to further enhance the extension force required to bring the fingers to the open hand state. The elastic modulus of the elastic fabric (0.45 N/mm) was chosen to match the elastic modulus of the elastic cords described in previous devices that allow the patients to flex their fingers voluntarily and provide assistance in the hand opening (Brokaw et al., 2011). The elastic cords are attached to the distal phalanx of each finger at the dorsal side. The tensions in the elastic cords increase with increasing finger flexion and pull the fingers to the open hand state. Assuming an offset distance of 10 mm due to skin stretching during finger flexion, the elastic fabric is able to provide an estimated 4.5 N of extension force to pull the fingers to the open hand state when the actuators are depressurized.

Pilot testing with two stroke survivors demonstrated the feasibility of the glove in providing functional grasp assistance for ADL. The glove is suitable for stroke survivors with a flaccid hand and without increased flexor tone (Modified Ashworth Scale >2). Without the assistance of the glove, the patients could not grasp cylindrical objects effectively, due to weak grasp strength and contracture. With the presence of the glove, the patients could then grasp the objects more effectively as the fingers were slightly more extended due to the elastic components of the glove. When the actuators were pressurized, the glove provided an additional grasping force to the patients' hand and assisted with the grasping activities. Therefore, the preliminary results showed that with the presence and assistance of the glove, the patients' grasping performance improved. The results of the USE questionnaire and the patients' feedback revealed that the patients were generally satisfied with the glove. Additionally, patients reported high level of comfort and ease of use. The parameter with the lowest rating is “Desire to purchase”. The patients pointed out that they would purchase if the selling price was within their affordable range, which was between 500 and 1,000 USD. Otherwise, they would prefer rental. The most positive aspects that the patients pointed out included (1) The glove serves the purposes of mobilizing the finger joints and assisting their grasping activities and (2) The glove motivates the patient to do rehabilitation exercises. On the other hand, the most negative aspect is that the fabric of the glove should be able to be sanitized and washed easily for hygiene purpose.

## Conclusion

This paper presented a soft robotic glove designed to assist stroke survivors with grasping tasks during their ADLs. Fabric-reinforced actuators with a corrugated fabric layer and a reinforced elastic layer were developed and experimental results have shown that the actuators could support finger motions with desired force output at lower operating air pressure, compared to the required operating pressure of previously developed actuators. A control system was built to allow isolated control of each actuator and integrated into a portable waist belt back. Both the glove and the control system are more lightweight than previously developed glove systems. The glove was evaluated with five healthy participants in terms of the assisted ROM and grip strength. Our results demonstrated that the glove was able to achieve sufficient ROM and provided assistance with motions typically found in ADL, in the absence of voluntary muscle control. A pilot test on two stroke survivors with reduced hand function also demonstrated that the glove allowed the patients to perform functional grasping activities more effectively.

## Future work

In this work, the experiments on grip force, glove-assisted ROM, and pilot tests with stroke survivors were conducted at a fixed actuator pressure (120 kPa). While the results from section Blocked Tip Force Output have shown that the actuator force increased with increased pressure, it is possible to conduct a more extensive study in the future by obtaining objective measurements, such as force, ROM, and grip strength, as a function of actuator pressure and presenting the time course of the pressure during each task. The force, ROM and grip strength can be controlled by adjusting the air pressure according to patient's condition. This feature will be further explored in subsequent work.

Future iterations of the glove will include sensor components, such as joint angle sensors and force sensors, which can provide joint angle and grasp force feedback. Joint angle feedback allows closed-loop position control and will enable the pump-valve control system to identify whether the desired hand-finger posture has been achieved. The grasp force feedback will permit us to examine the force output of the glove and to facilitate impedance control strategies (Marchal-Crespo and Reinkensmeyer, 2009).

While stroke survivors are the target patient population of the glove presented in this work, the glove can also potentially assist patients with incomplete spinal cord injuries or amyotrophic lateral sclerosis. Apart from serving as an assistive device for ADLs, the glove described in this work can potentially serve as a rehabilitative device, providing task-specific rehabilitation training. Several research groups working on hand exoskeletons have shown that incorporation of these devices into hand therapy can benefit the stroke survivors. Significant improvements in functional outcome have been observed with the combination of assist-as-needed control strategy and user intent detection strategy (Hu et al., 2013; Thielbar et al., 2014, 2017). For example, Theilbar et al. have developed a cable-driven robotic glove with the combination of voice and EMG to detect user intention. Their study has shown that incorporation of the glove into the rehabilitation therapy has the potential for clinical use to improve hand function.

For stroke patients with increased flexor tone and spasticity (Modified Ashworth Scale >2), the passive extension mechanism might not have enough force to extend the fingers. For these patients, soft robotic glove that is capable of providing active flexion and extension will be preferable. Finally, a larger clinical study will be conducted in order to evaluate and improve the actuator and glove design, and to study the long-term efficacy of the glove-assisted intervention in rehabilitation.

## Ethics statement

This study was carried out in accordance with the recommendations of Institutional Review Board of the National University of Singapore (B-14-141) and the NHG Domain Specific Review Board (2015/00975) with written informed consent from all subjects. All subjects gave written informed consent in accordance with the Declaration of Helsinki. The protocol was approved by the Institutional Review Board of the National University of Singapore (B-14-141) and the NHG Domain Specific Review Board (2015/00975).

## Author contributions

HY, JL, FN, and CY designed the study. HY collected, processed and analyzed the data and drafted the manuscript. JL, FN, and CY contributed to the interpretation of findings. CY oversaw its coordination and helped to draft the manuscript. All authors read, edited and approved the final manuscript.

### Conflict of interest statement

The authors declare that the research was conducted in the absence of any commercial or financial relationships that could be construed as a potential conflict of interest.
