# Corrigendum: Design and Preliminary Feasibility Study of a Soft Robotic Glove for Hand Function Assistance in Stroke Survivors

**DOI:** 10.3389/fnins.2018.00323

**Published:** 2018-05-08

**Authors:** Hong Kai Yap, Jeong Hoon Lim, Fatima Nasrallah, Chen-Hua Yeow

**Affiliations:** ^1^Department of Biomedical Engineering, National University of Singapore, Singapore, Singapore; ^2^NUS Graduate School for Integrative Sciences and Engineering, National University of Singapore, Singapore, Singapore; ^3^Department of Medicine, National University of Singapore, Singapore, Singapore; ^4^Queensland Brain Institute, University of Queensland, St. Lucia, QLD, Australia

**Keywords:** soft robotic, hand exoskeleton, rehabilitation, activities of daily living, soft actuators

In the original article, there was an error in the stated funding support. The correct Funding Statement appears below.

The authors apologize for this oversight. This error does not change the scientific conclusions of the article in any way.

The original article has been updated.

## Conflict of interest statement

The authors declare that the research was conducted in the absence of any commercial or financial relationships that could be construed as a potential conflict of interest.

