# Supplementary material for: Design and Preliminary Feasibility Study of a Soft Robotic Glove for Hand Function Assistance in Stroke Survivors
Source: Front Neurosci. 2017 Oct 9;11:547. doi: 10.3389/fnins.2017.00547 (PMC5640819; doi:10.3389/fnins.2017.00547)
Supplement: Supplementary file 1 [file Presentation1.PDF]

Supplementary Material

Design and Evaluation of a Soft Wearable Robotic Glove for Hand Function Assistance in Stroke Survivors using Fabric-Reinforced Soft Pneumatic Actuators

Hong Kai Yap, Jeong Hoon Lim, Fatima Nasrallah, and Chen-Hua Yeow*

*** Correspondence:** Chen-Hua Yeow: rayeow@nus.edu.sg

1 Comparison of Soft Actuators

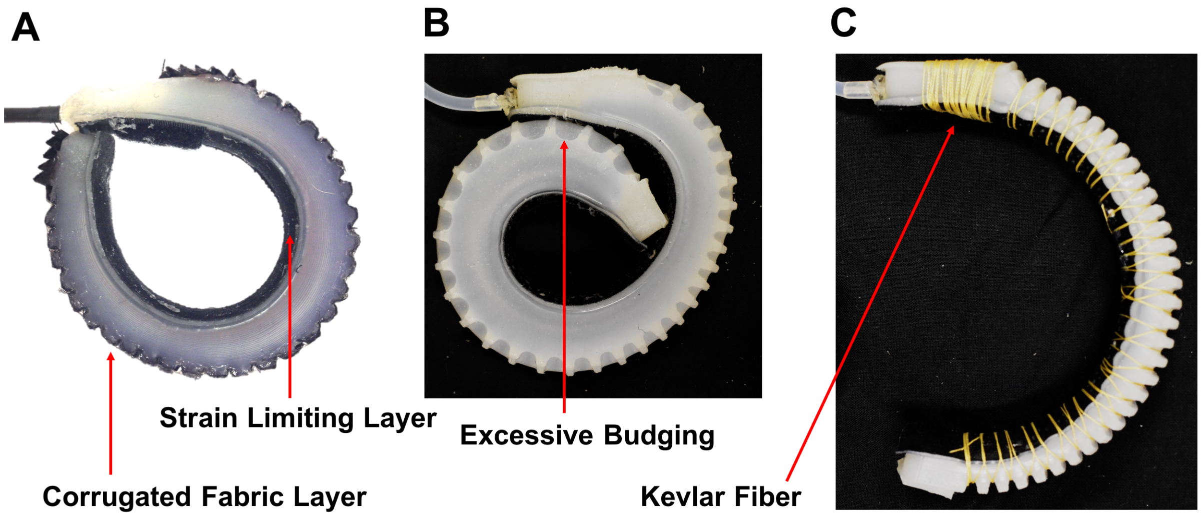

Figure S1. Comparison of different soft actuators, A) Fabric-reinforced elastomeric actuator, B) Elastomeric actuator without any reinforcement and C) Fiber-reinforced elastomeric actuator.

Figure S1 shows the comparison of different soft actuators. The actuators were of similar dimension and pressurized at the same pressure, 120kPa. Without any reinforcement, the bending capability of the actuator was the highest (i.e. the bend radius of the actuator was the smallest). However, excessive radial budging could be observed, especially at the distal part, which increased the vulnerability to rupture in the walls. With fiber-reinforcement, the radial budging was effectively constrained. However, the bending capability of the actuator was the lowest. As a result, higher pressure will be required to achieve desired bending motion.

2 Fabrication Process of Soft Actuator

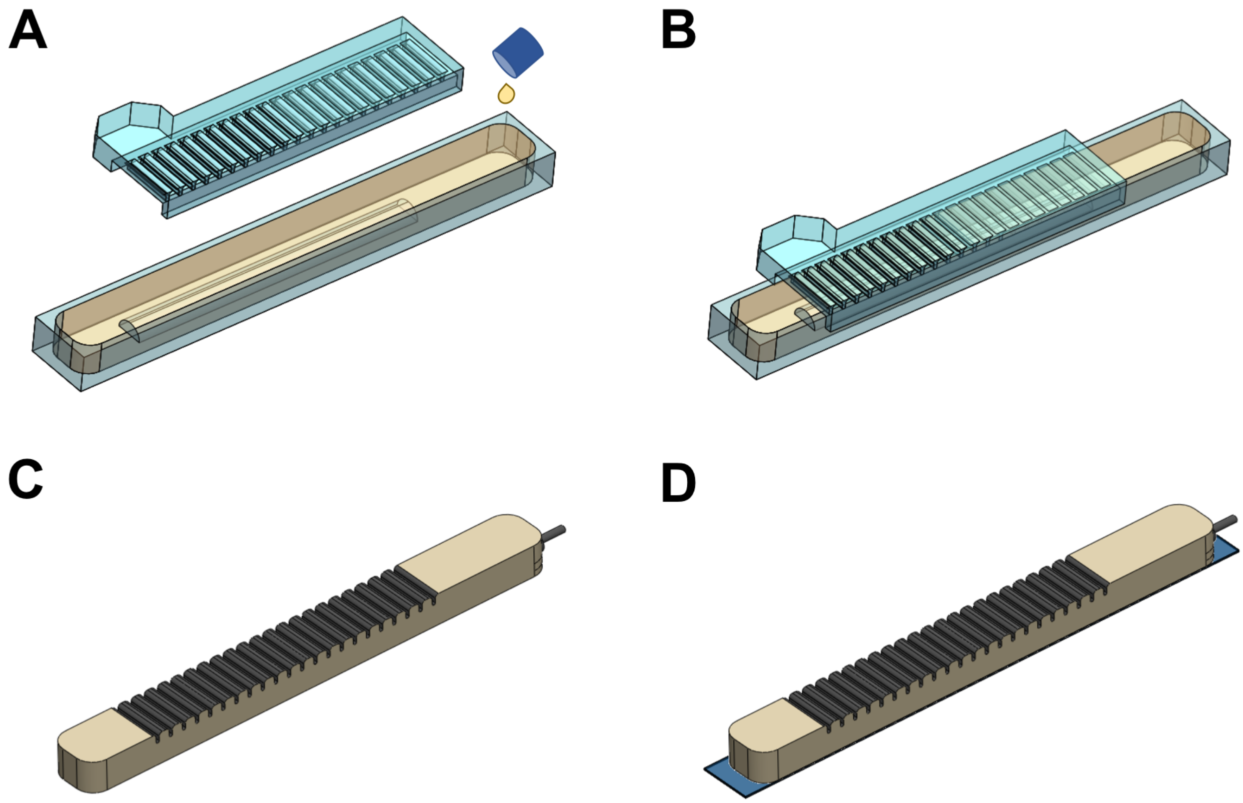

Figure S2. Fabrication process of the actuator, A) Pour liquid elastomer to the chamber mold, B) Place the top layer mold on top to create corrugated outer layer, C) The ensemble is cured under 60˚C. A pneumatic adapter is inserted, D) A fabric was attached to the corrugated outer layer. Seal the bottom of the cured structure with an elastic fabric.

3 Mold Dimension

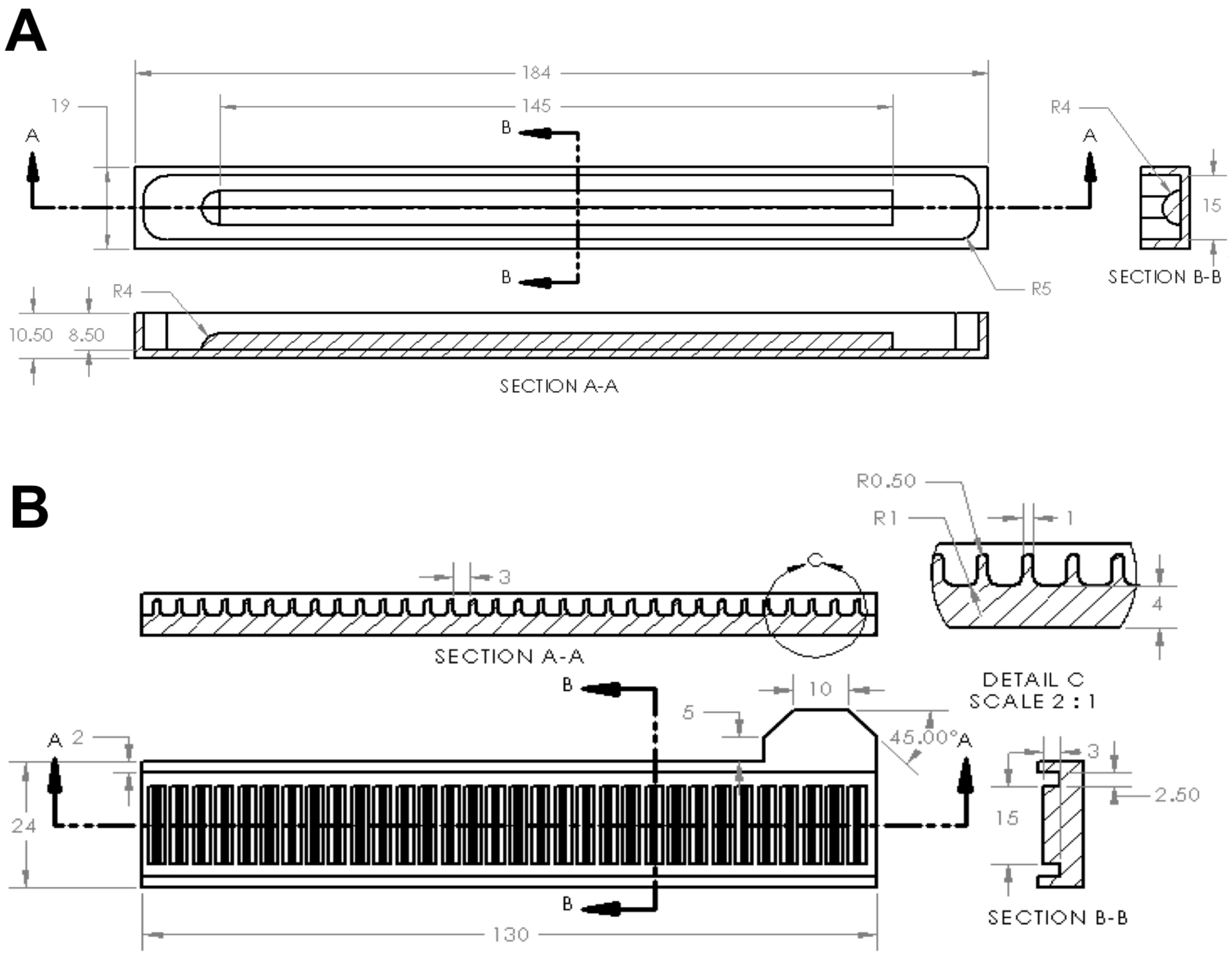

Figure S3. Dimension of the A) chamber and B) top layer molds.

4 Theoretical Model of Soft Actuator

To obtain the blocked tip force values, the actuator was constrained in a flat configuration. With this configuration, the actuator's height, curvature, and bending angle were constrained during pressurization. To estimate the tip force in this configuration, Polygerinos *et. al.* (Polygerinos et al., 2015) and Wang *et.al.* (Wang et al., 2016) have derived an expression by extending the analytical model that was previously developed for fiber-reinforced actuators.

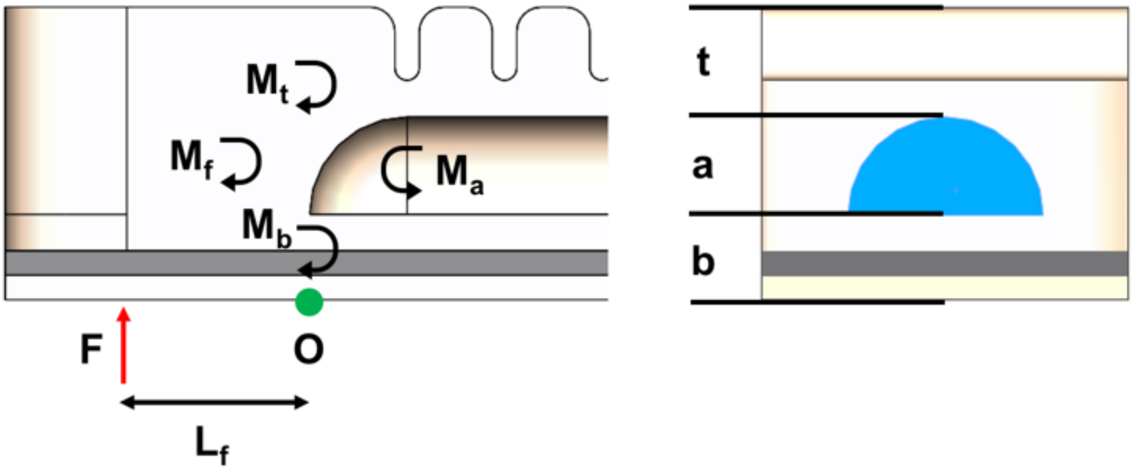

Figure S4. Side view of the actuator tip showing the generated moments and cross-sectional view of the actuator.

The moment equilibrium at the pivot point O needs to satisfy to following condition:

$$\boldsymbol{M_a = M_f + M_t + M_b}$$

where

$\boldsymbol{M_a}$: Pressure-induced bending moment generated by the internal air pressure acting on the actuator tip around the pivot point O.

$\boldsymbol{M_f}$: Tip force bending moment generated by the interaction force applied to the actuator tip when the actuator is in contact with an external object, in our case, the load cell.

$\boldsymbol{M_t}$: Material stretch moment of the top layer of the actuator

$\boldsymbol{M_b}$: Material stretch moment of the bottom layer of the actuator

Since the actuator was constrained in zero bending angle, we assume that the material stretch moments are close to zero. Therefore,

$$\boldsymbol{M_t = M_b = 0}$$

For actuator with hemi-circular air chamber with radius a, the pressure-induced bending moment M_a can be calculated as (Polygerinos et al., 2015):

$$\boldsymbol{M_a = \frac{1}{6}(4a^3 + 3\pi a^2 b)P_{in}}$$

where

$\boldsymbol{P_{in}}$**:** Input Air Pressure

$\boldsymbol{a}$**:** Radius of the hemi-circular air chamber

$\boldsymbol{b}$**:** Thickness of the bottom layer of the actuator

In the model presented by Polygerinos *et. al.* (Polygerinos et al., 2015) and Wang *et.al.* (Wang et al., 2016), they assumed that the cross-sectional area of the air chamber remained constant due to the constraint of fiber reinforcement. In our case, the cross-sectional area of the air chamber increased initially and further constrained by the fabric-reinforcement layer. Therefore, we assume that the radius, a increases with increasing pressure and begins to plateau at high pressure. The relationship between the radius, a and the pressure, P_{in} was obtained empirically. The perimeters of the actuator at different air pressures were measured using a flexible thread. Assuming the wall thickness of the actuator remains constant, the radius, a at certain pressure can be obtained. The expression of the relationship between the radius, a and the pressure, P_{in} was then obtained through least squares quartic polynomial fitting.

$$\boldsymbol{a = -5 \cdot 10^{-23} {P_{in}}^4 + 1 \cdot 10^{-17} {P_{in}}^3 - 4 \cdot 10^{-13} {P_{in}}^2 + 1 \cdot 10^{-8} P_{in} + 5 \cdot 10^{-7}}$$

Therefore, the tip force, F can be obtained.

$$\boldsymbol{F = \frac{M_f}{L_f} = \frac{M_a}{L_f}}$$

where

$\boldsymbol{L_f}$**:** The distance between the pivot point, O and the load cell tip.

5 Bandwidth of Soft Actuator

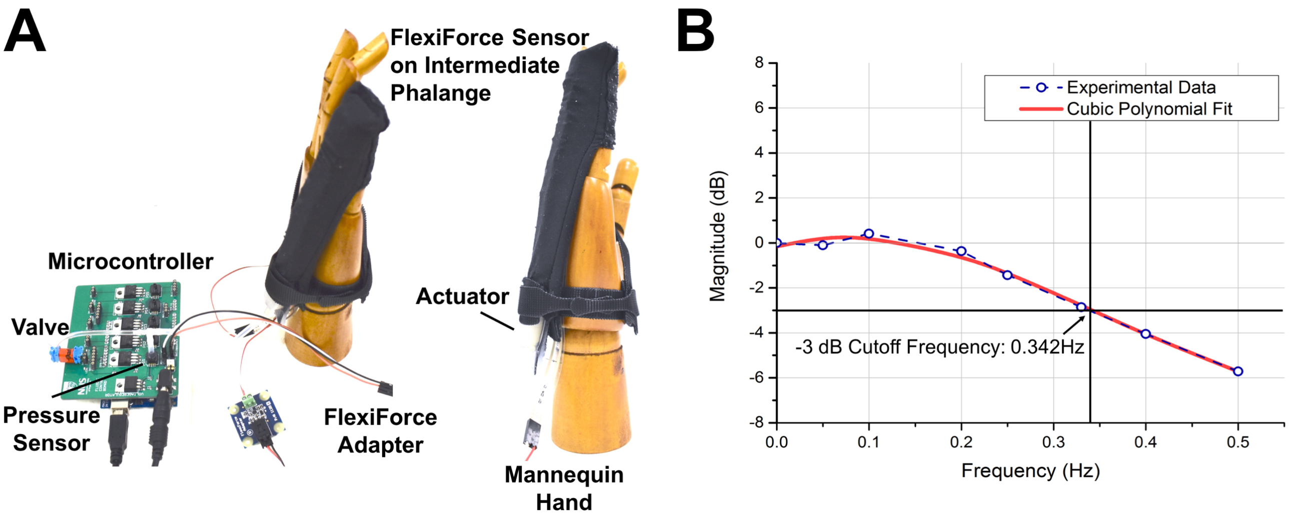

Figure S5. A) Setup for bandwidth experiment. B) Frequency response of an actuator to sinusoidal input.

To determine the bandwidth of the actuator, a sinusoidal input pressure with amplitude of 120kPa was applied to the actuator and modulated at varying frequencies. A FlexiForce (A201, Tekscan Inc, USA) sensor was secured on the intermediate phalange to measure the applied force of the actuator on the finger of a mannequin hand. Figure S6b shows the frequency response of an actuator to the sinusoidal input. The -3dB bandwidth was found to be around 0.342Hz.

6 Test Method for Glove-assisted Range of Motion

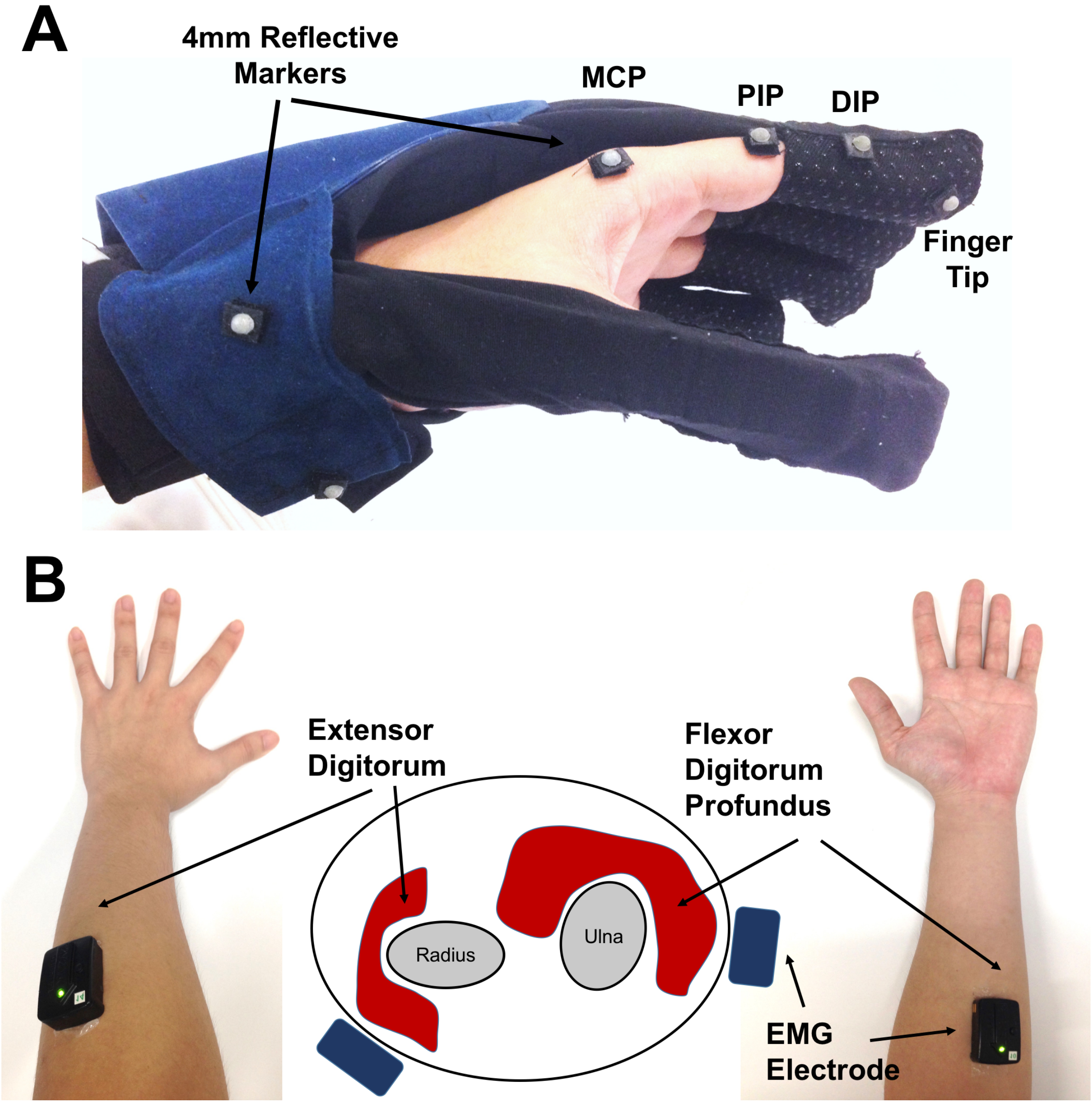

Figure S6. Placement of the (A) reflective markers and the (B) EMG electrodes.

An optical-based motion analysis system with eight cameras (T-Series, T160, Vicon Motion System Ltd., UK) was used to capture the assisted-finger range of motion. Four reflective markers were attached to the glove at locations corresponding to the fingertip, distal interphalangeal (DIP), proximal interphalangeal (PIP) and metacarpophalangeal (MCP) joints of the index finger (Fig. S6A). The marker placement was identified by rotating each finger segment of its joint center.

The motion profile of the index finger was tracked in this case as the index finger provided the best line of sight for the cameras to detect the markers. The trajectories of the markers were recorded by the cameras at a sampling rate of 100Hz. Two wireless electromyography (EMG) electrodes (Trigno,

Delsys Inc, USA) were placed on two muscle pairs of the finger flexors and extensors (namely flexor digitorum profundus and extensor digitorum) and secured to the skin by a double-sided adhesive interface (Fig. S6B). Raw EMG signals were collected at 1000 Hz. The EMG signals during maximal voluntary contractions were collected prior to the actual session.

The EMG data was first normalized with respect to maximal voluntary contractions. The kinematics and EMG data were averaged across three trials for each subject and further averaged across five participants and analyzed using Matlab (MathWorks, USA) with customized code to calculate the range of motion of each joint as well as the differences of EMG signals between active and glove-assisted sessions. Both kinematics and EMG signals were time normalized to one full exercise cycle (hand closing followed by opening). EMG signals were first full wave rectified and then, linear envelopes of the signals were created using a 2nd order Butterworth low pass filter with a cut-off value of 8Hz.

7 Phone Application

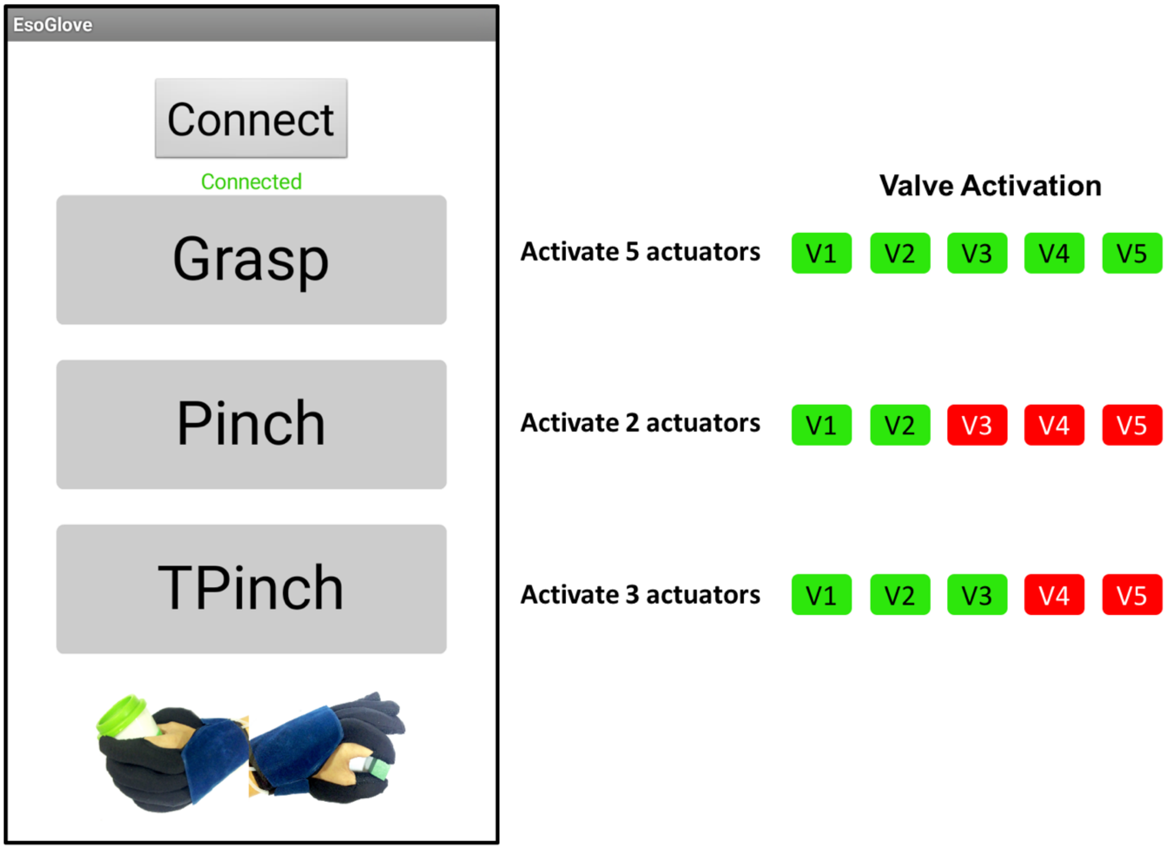

Figure S7. Screenshot of the phone application interface with three virtual buttons that corresponded to grasping, pinching, and tripod pinching. Activation of specific valves was pre-programmed in the microcontroller. The application communicated with the microcontroller via Bluetooth.

References

Polygerinos, P., Wang, Z., Overvelde, J. T. B., Galloway, K. C., Wood, R. J., Bertoldi, K. & Walsh, C. J. 2015. Modeling of Soft Fiber-Reinforced Bending Actuators. *IEEE Transactions on Robotics,* PP**,** 1-12.

Wang, Z., Polygerinos, P., Overvelde, J., Galloway, K., Bertoldi, K. & Walsh, C. 2016. Interaction Forces of Soft Fiber Reinforced Bending Actuators. *IEEE/ASME Transactions on Mechatronics,* PP**,** 1-1.
